# Current Advancement on the Dynamic Mechanism of Gastroesophageal Reflux Disease

**DOI:** 10.7150/ijbs.65066

**Published:** 2021-10-03

**Authors:** Zhi Zheng, Yuxi Shang, Ning Wang, Xiaoye Liu, Chenglin Xin, Xiaosheng Yan, Yuhao Zhai, Jie Yin, Jun Zhang, Zhongtao Zhang

**Affiliations:** 1 Department of General Surgery, Beijing Friendship Hospital, Capital Medical University, Beijing, China.; 2 Beijing Key Laboratory of Cancer Invasion and Metastasis Research, Beijing, China.; 3 National Clinical Research Center for Digestive Diseases, Beijing, China.; 4 Beijing Institute of Clinical Medicine, Beijing, China.; 5 Department of Hematology, Fuxing Hospital, Eighth Clinical Medical College, Capital Medical University, Beijing, China.; 6 Department of Anesthesiology, Beijing Friendship Hospital, Capital Medical University, Beijing, China.

**Keywords:** gastroesophageal reflux disease, dynamic mechanism, anti-reflux barrier disruption, esophageal clearance impaired, advancement

## Abstract

Gastroesophageal reflux disease (GERD) is a common clinical disease associated with upper gastrointestinal motility disorders. Recently, with improvements in living standards and changes in lifestyle and dietary habits, the incidence of GERD has been increasing yearly. However, the mechanism of GERD has not been fully elucidated due to its complex pathogenesis, and this had led to unsatisfactory therapeutic outcomes. Currently, the occurrence and development of GERD involve multiple factors. Its pathogenesis is mainly thought to be related to factors, such as lower esophageal sphincter pressure, transient lower esophageal sphincter relaxation, crural diaphragmatic dysfunction, hiatus hernia, and impaired esophageal clearance. Therefore, explaining the pathogenesis of GERD more clearly and systematically, exploring potential and effective therapeutic targets, and choosing the best treatment methods have gradually become the focus of scholars' attention. Herein, we reviewed current advancements in the dynamic mechanism of GERD to better counsel patients on possible treatment options.

## Introduction

Gastroesophageal reflux disease (GERD) is one of the most common upper gastrointestinal tract diseases, which mainly results in acid reflux, heartburn, intractable cough, and asthma caused by reflux of upper gastrointestinal contents into the esophagus 1, 2. As the disease progresses, some patients may develop esophagitis, esophageal ulcers, and esophageal stenosis. In severe cases, GERD can lead to Barrett's esophagus or esophageal cancer, which can affect a patient's quality of life and long-term prognosis 3. Recently, the incidence of GERD has been increasing yearly due to improvements in living standards and changes in lifestyle and dietary habits of people 4, 5. However, the pathogenesis of GERD and other different types of dynamic disorders has not yet been fully elucidated, and this has led to unsatisfactory therapeutic outcomes 6, 7. Therefore, explaining the pathogenesis of GERD more clearly and systematically, exploring potential and effective therapeutic targets, and selecting the best treatment have gradually become the focus of scholars' attention 8. Currently, the pathogenesis of GERD is generally believed to be related to reduction in the pressure of the lower esophageal sphincter (LES), transient and excessive lower esophageal sphincter relaxation (TLESR), hiatal hernia, esophageal clearance dysfunction, acid pockets, esophageal hypersensitivity, and mucosal barrier damage 9-14. Meanwhile, an in-depth study of the pathogenesis of GERD would aid in promoting the progression of its diagnosis and treatment, reducing its incidence, and improving the quality of life and prognosis of patients 15. Therefore, the purpose of this study was to systematically review current research advancements on the dynamic mechanism of GERD, with the aim of providing a reference for clinical practice.

## Methods

We conducted a literature search for published manuscripts on GERD up to August 2021 in PubMed, Web of Science, and EMBASE databases, and employed the following search terms: “gastroesophageal reflux disease,” “reflux esophagitis,” “epidemiology,” “pathogenetic mechanism,” “dynamic mechanism, “esophageal motility disorder,” “lower esophageal sphincter pressure reduction,” “esophageal clearance dysfunction,” “hiatal hernia,” and “transient lower esophageal sphincter relaxation.” Qualitative and quantitative data were extracted by interpreting each paper in cycles to avoid missing potentially valuable data.

## Epidemiology of GERD

**Figure 1:**
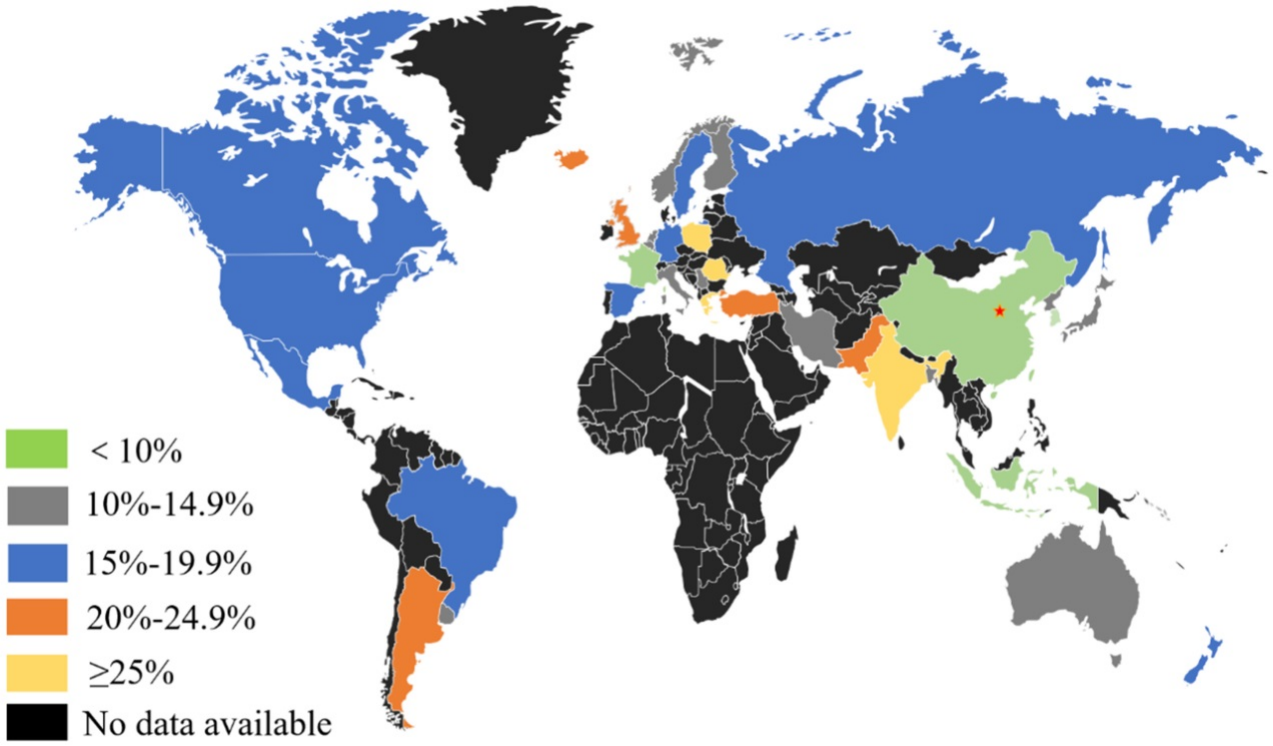
Incidence of GERD symptoms worldwide. Generally, the prevalence rate is relatively high in developed western countries and lower in Asia.

GERD is a disease whose treatment requires substantial medical resources 16, and its risk factors include smoking, obesity, alcohol consumption, non-steroidal anti-inflammatory drugs (NSAIDs), social factors, psychosomatic diseases, and genetic factors 17-21. Meanwhile, there are also correlation between GERD and ethnicity, Helicobacter pylori infection, gender, age, and lifestyle 22-25. A study reported that the prevalence of GERD varies greatly in different countries and regions, and its clinical characteristics, etiology, and pathogenesis factors vary 26. Previous studies have conducted relevant epidemiological investigations on the clinical symptoms and incidence of GERD in different countries and regions 27-31 **(Table 1)**. Overall, the prevalence rate of GERD is relatively high in developed western countries and is low in Asia. Moreover, the severity of the disease in Asia is relatively milder than that in western countries 32-44 **(Figure 1)**. A meta-analysis showed that the prevalence of GERD in western countries is approximately 10-20%, while that in Asian countries is <10% 17.The global incidence of GERD has been increasing annually. Studies have reported that the incidence of GERD in North America is as high as 27.8%, and is 25.9% in Europe 8, 45, 46. In addition, Daniele et al. investigated 137,081 cases from 2005 to 2014 and found that the average annual incidence of GERD was 101.3/10,000, which seriously affected people's quality of life and mental state. This is a clinical problem that needs to be solved urgently 47. However, there are relatively few studies on the incidence of GERD. Most of which are only epidemiological surveys conducted in certain regions. Moreover, clinical studies based on the characteristics of the Chinese population are unavailable, and this has led to limitations in the value of the clinical diagnosis and treatment of GERD. Based on this, a large-scale domestic epidemiological survey involving 16,078 patients was conducted. The results showed that the rate of heartburn and/or reflux at least once a week was 5.2% (3.2-7.5%), the prevalence of heartburn at least once a week was 1.8%, the incidence of reflux at least once a week was 4.2%, and the incidence of GERD that met the Montreal definition was 3.1% (1.7-5.1%) 48. However, the limitation of this study is that clinical data on new onset cases of GERD in Chinese populations were not obtained 48. Hence, it is necessary for scholars to carry out a larger epidemiological investigation in the future to provide a reference for clinical practice.

## Pathogenetic mechanism of GERD

Usually, the anti-reflux defense mechanism of the esophagus and the erosive effect of refluxed substance on the esophageal mucosa are in equilibrium. When the former's defense mechanism is reduced or the latter's injurious effect is enhanced, the balance is broken, and this may lead to the occurrence of GERD 49. The primary pathophysiological mechanisms of GERD are anti-reflux barrier function weakening and impaired esophageal clearance function 50, 51.

### Anti-reflux barrier disruption at the esophageal junction

#### Hypotensive lower esophageal sphincter pressure

**Figure 2:**
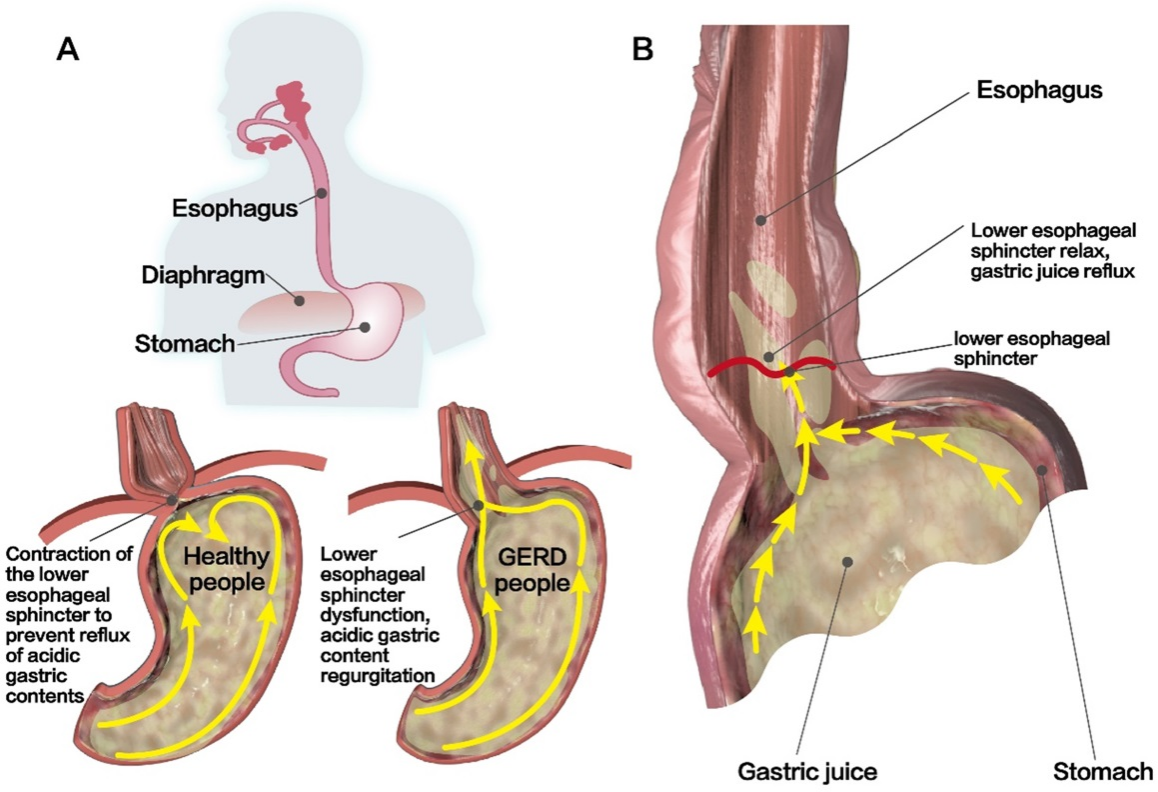
** Structural characteristics of the LES. (A)** In healthy people, LES contraction can lead to an increased LES pressure that can prevent acidic gastric contents from reflux. However, LES dysfunction in patients with GERD can lead to acidic gastric content regurgitation. **(B)** Reflux occurs when the resting pressure of LES is abnormally low, resulting in higher gastric pressure than esophageal pressure.

**Figure 3:**
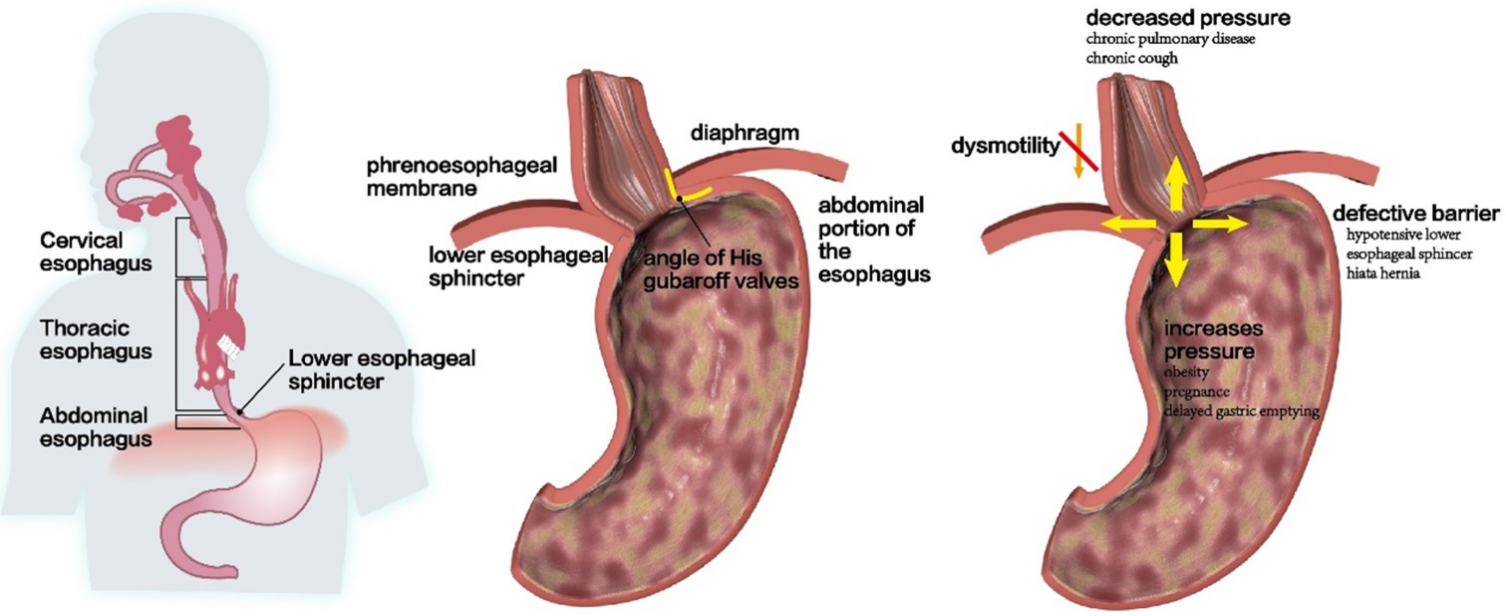
** Anti-reflux barrier disruption at the esophageal junction. (A and B)** In healthy people, the resting pressure of LES, an anti-reflux barrier at the esophagogastric junction, is approximately 10-30 mmHg. **(C)** People with obesity, pregnancy, or gastric emptying disorders had significantly higher gastric pressure and average gastroesophageal pressure gradient, which promoted the GERD occurrence. In addition, hypotensive LES, hiatal hernia, and dysmotility are also related to GERD occurrence.

The LES is the most important structure of the anti-reflux barrier at the esophagogastric junction 52, 53. After eating, LES relaxation in healthy people leads to a decrease in LES pressure, which is conducive for the digestion of food in the gastric cavity. At night or when sleeping, LES contraction leads to an increase in LES pressure and this prevents reflux 52, 54** (Figure 2A)**. Therefore, the pressure difference between the esophagus and the gastric cavity is an important factor in the prevention of reflux. Reflux occurs when the resting pressure of the LES is abnormally low, resulting in a gastric pressure that is higher than the esophageal pressure 55 **(Figure 2B)**. In healthy people, the resting pressure of the LES is approximately 10-30 mmHg, as this is a high-pressure zone that is formed by contraction of the LES, which can prevent the reflux of gastric contents and bile into the esophagus 56 **(Figure 3A and 3B)**. In addition, high-resolution esophageal manometry (HRM) has shown that the LES pressure of patients with GERD, especially those with reflux esophagitis, was significantly lower than that of healthy subjects 57, 58. Compared to healthy subjects, individuals with obesity, pregnancy, or gastric emptying disorders have been shown to have significantly higher gastric pressure and average gastroesophageal pressure gradient, which may also promote the occurrence of GERD 59-61** (Figure 3C)**. In addition, another study conducted pH monitoring on 310 patients with GERD for two consecutive days and found that 83% of the patients with abnormal pH values for 2 days had LES damage. Moreover, LES damage was also observed in 35% of the patients with abnormal pH levels for only 1 day and in 17% of those with normal pH values for 2 days, suggesting that the incidence of GERD was related to LES damage 62. This may be mainly attributed to the shortened length of LES and decreased resting pressure of LES, which is positively correlated with the degree of esophageal acid exposure. Recent studies have also found that changes in parameters, such as low LES pressure score or low esophagogastric junction (EGJ) contraction integral in high-resolution esophageal manometry may also be closely related to the occurrence of gastroesophageal reflux 15, 63, 64; however, the value of its clinical application remains to be further explored.

The treatment of GERD mainly includes drug treatment, endoscopic treatment and surgical treatment. In addition to drug treatment, surgical treatment has become the primary therapy 1. The key to surgery is to fold the fundus of the stomach to surgically restore the structure of the EGJ, the normal anatomical position of the LES, and the length of the LES, through proper suture fixation. Adequate restoration and maintenance of the anti-reflux function of the LES, is required for surgical treatments to achieve a suitable unity of structure and function 65.

#### Transient lower esophageal sphincter relaxation

**Figure 4:**
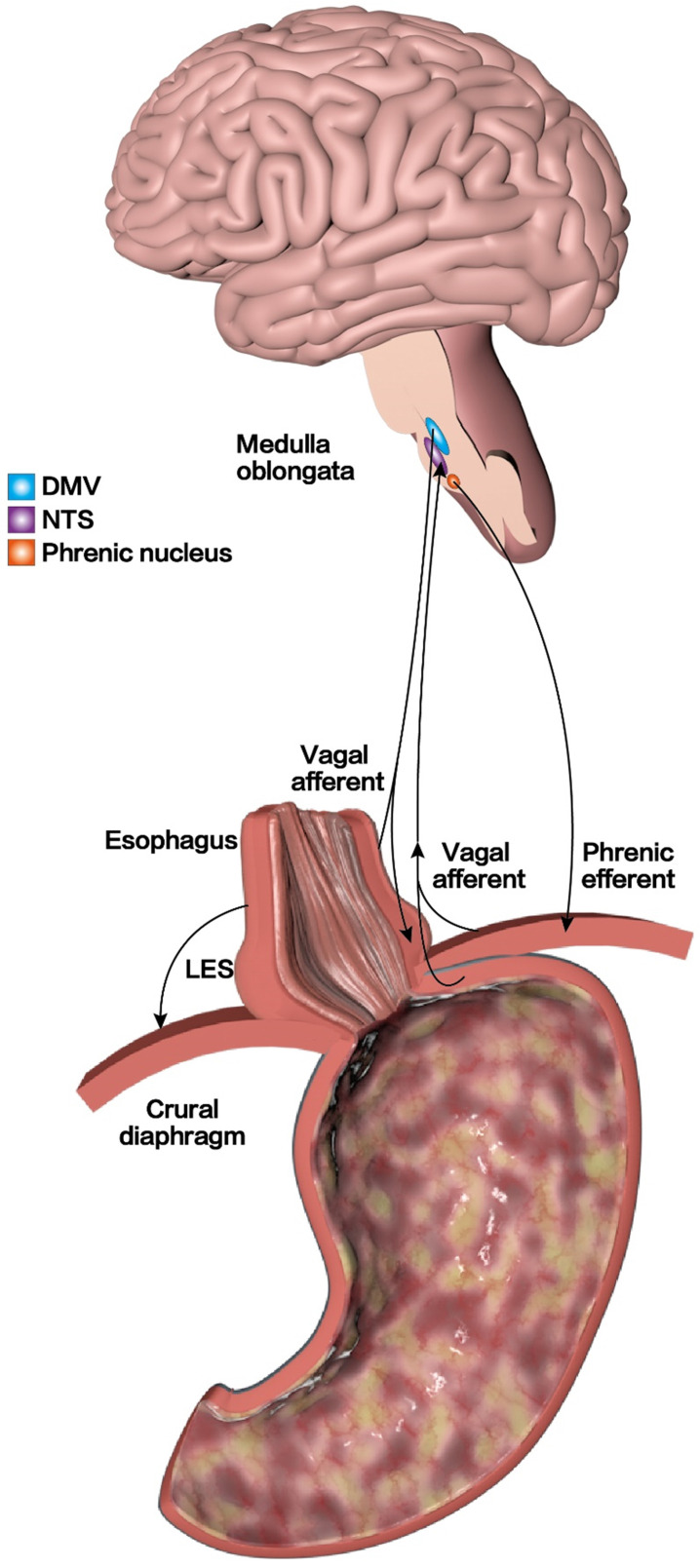
** TLESR mechanism of GERD.** In the case of gastric distention, the vagal afferent fibers around EGJ are activated, and the nerve impulses after activation are transmitted along the vagal afferent fibers to the solitary tract nucleus. Subsequently, it triggers the signal transmission between the nucleus of the solitary tract (NTS) and the dorsal motor nucleus of the vagus nerve (DMV), and finally nerve impulses are transmitted along the efferent fibers of the vagus to the LES and crural diaphragm, weakening the role of the anti-reflux barrier and causing the onset of GERD.

With an in-depth study of the pathogenesis of GERD, it is now generally believed that TLESR is the main cause of gastroesophageal reflux 66. TLESR refers to the transient spontaneous relaxation of the LES without swallowing. The relaxation time can last for 10-45 s or more, which is often accompanied by reflux of gastric and duodenal fluids 8, 67 **(Figure 2B)**. Although TLESR can occur in healthy people with normal LES pressure or in patients with GERD, the refluxed content is different. The former present mostly with gas reflux and most cases have no obvious reflux symptoms, while the latter often presents with acid reflux, which is associated with chronic reflux diseases in most cases 50, 68, 69. Studies have found that only 40-50% of TLESR are associated with acid reflux in healthy people, compared to 60-70% in patients with GERD 70, 71. Another studies have found that the frequency and duration of TLESR were significantly higher in patients with GERD than in healthy participants, which confirmed that TLESR is an important mechanism that causes GERD reflux symptoms 72. The mechanism of GERD caused by TLESR may be related to changes in EGJ compliance and the increase in EGJ pressure gradient 73. In addition, the esophagus will continue to shorten during TLESR due to longitudinal esophageal muscle contraction, thereby promoting reflux 74, 75. Meanwhile, gastric distention and intractable constipation that cause increased abdominal pressure can also lead to TLESR 70. To further explore the potential pathogenesis of TLESR, it has been reported that TLESR is a mode of vagus-vagus-mediated conduction 52, 76. In the case of gastric distention, the vagal afferent fibers around the EGJ are activated and the nerve impulses after activation are transmitted along the vagal afferent fibers to the solitary tract nucleus. This subsequently triggers the transmission of signals between the nucleus of the solitary tract (NTS) and the dorsal motor nucleus of the vagus nerve (DMV); finally, nerve impulses are transmitted along the efferent fibers of the vagus to the LES and crural diaphragm, which induces relaxation of the LES, esophageal shortening, and decreased tension of the crural diaphragm. Thus, weakening the role of the anti-reflux barrier and causing the onset of GERD 52, 76** (Figure 4)**. However, studies have confirmed that γ-aminobutyric acid receptor agonists have inhibitory effects on afferent vagal signals, signal transmission between the NTS and DMV, and efferent vagal signals. Therefore, to treat GERD, studies have suggested reducing the frequency of TLESR 51, 77, 78. Animal experiments show that Baclofen, a GABA-B receptor agonist, can effectively prevent the occurrence of TLESR and reflux 79, 80. Moreover, clinical studies have also confirmed that Baclofen can significantly reduce TLESR in both healthy volunteers and patients with GERD and effectively inhibit acid reflux 81-83. Therefore, the activation of GABA-B receptors can be used as a therapeutic target to inhibit the excitation of the vagus nerve and reduce the occurrence of TLESR. However, its clinical application has been limited because of the adverse reactions and the short half-life of Baclofen 84. Therefore, researchers need to further search on potential therapeutic targets to inhibit TLESR and better benefit patients with GERD.

#### Crural diaphragmatic dysfunction

The EGJ pressure is mainly composed of the crural diaphragm tension and LES pressure. Among them, the crural diaphragm tension is affected by breathing movement, while LES tension is affected by swallowing movement 85. Therefore, it is necessary to comprehensively consider the influence of breathing and swallowing when calculating the EGJ pressure** (Figure 3B)**. It has been found that the end-expiratory pressure of the EGJ comes from the LES, while the end-inspiratory pressure comes from the crural diaphragm tension under normal circumstances. Therefore, the abnormal anatomy and function of the crural diaphragm is another important factor that causes gastroesophageal reflux. A previous study found that at rest, the crural diaphragm tension of patients with GERD is significantly lower than that of healthy participants. Moreover, the magnitude of the reduction in tension is positively correlated with the degree of acid reflux, which indicates that a reduced crural diaphragm tension may directly weaken the anti-reflux barrier effect of LES 86. However, due to the complex anatomical structure and function of the EGJ, evaluation of its anti-reflux barrier function needs to consider the effect of the LES, diaphragm, and respiratory cycle, which is inconvenient for clinical diagnosis and treatment. Therefore, with the development of HRM, the esophageal-gastric junction contraction index (EGJ-CI), a new parameter, was used in the comprehensive evaluation of EGJ's anti-reflux barrier ability. Its advantage is that it integrates the changes in EGJ respiration, LES length, and pressure, and simplifies the function analysis of the EGJ 87. Studies have confirmed that there is a significant difference in EGJ-CI between patients with GERD and those of healthy subjects, and this value can reflect the changes in the anti-reflux barrier function of patients with GERD 88. Jasper et al. performed HRM and 24-hour pH impedance detection in patients with GERD and in healthy subjects, and found that EGJ-CI was a new parameter that could best summarize EGJ barrier function in the whole HRM measurement. This indicates that EGJ-CI may be the best parameter that can be used to predict pathological reflux. It is also a new indicator of EGJ contraction over time 89. The assessment of the EGJ barrier function can be improved to some extent. Additionally, another study found that when the cut-off value of EGJ-CI was 30, the sensitivity and specificity of EGJ-CI in predicting the occurrence of GERD were 77.8% and 81.7%, respectively, indicating that EGJ-CI had good accuracy and specificity in predicting GERD. This further proved the importance of anti-reflux barrier function in the pathogenesis of GERD 90. Based on this, EGJ-CI was used as a new parameter to reflect the anti-reflux barrier ability of the EGJ in the 3^rd^ edition of the Chicago classification, and it has been widely adopted by scholars gradually 87.

#### Hiatal hernia

**Figure 5:**
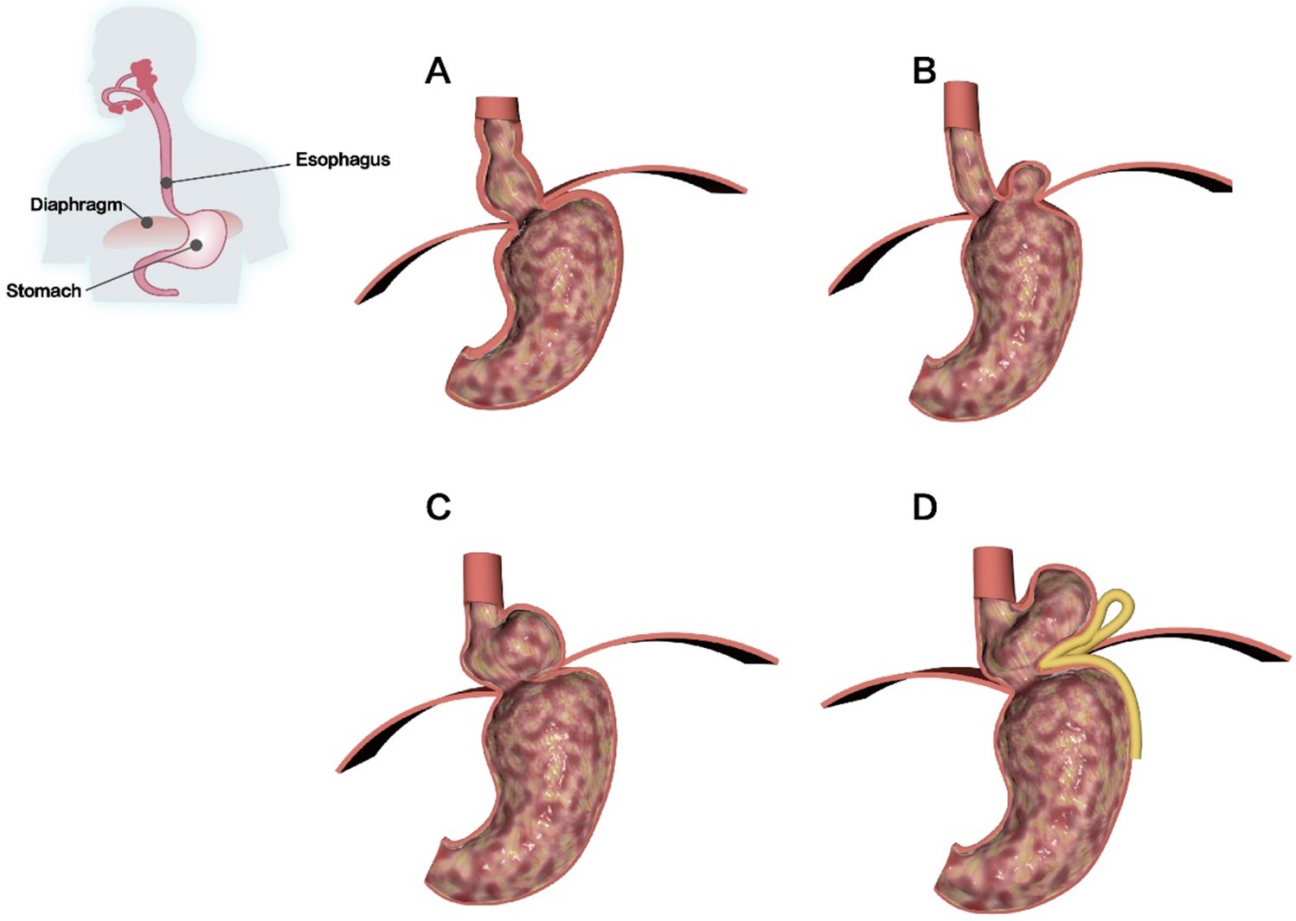
** Classification of HH. (A)** Type I is a sliding HH with a small hernia sac. **(B)** Type II is a para-hiatal hernia in which the fundus of the stomach can enter the thoracic cavity through the hiatus. **(C)** Type III is a mixed HH. The esophageal hiatus has large defects, but no other organ in the abdominal cavity can enter the thoracic cavity. **(D)** Type IV is a giant HH in which the stomach and other organs pass through the esophageal hiatus and enter the thoracic cavity due to the defect of the huge esophageal hiatus.

The formation of hiatal hernia (HH) is the main cause of structural abnormalities at the EGJ, among which the increase in intra-abdominal pressure caused by various reasons is the most common cause. HH is a disease caused by temporary or permanent entry of abdominal organs or tissue through the esophageal hiatus of the diaphragm into the thoracic cavity. GERD with HH is commonly seen in clinical settings 10, 91, 92. HH can be divided into the following four types: 1) type I is a sliding HH with a small hernial sac; 2) type II is a parahiatal hernia in which the fundus of the stomach can enter the thoracic cavity through the hiatus; 3) type III is a mixed HH, which has the characteristics of type I and type II HH. The esophageal hiatus has large defects, but no other organs in the abdominal cavity can enter the thoracic cavity; and 4) type IV is a giant HH that is similar to a type III HH. In this type, the stomach and other organs pass through the esophageal hiatus and enter the thoracic cavity due to a defect in the huge esophageal hiatus 93, 94** (Figure 5)**. Among them, type I HH is the most common. However, most patients can remain asymptomatic for life and if symptoms occur, it is likely to be gastroesophageal reflux 95, 96. Types II, III, and IV HH are rare, but they often cause severe clinical symptoms and require surgical intervention; therefore, they have important clinical significance 95, 96.

Studies have confirmed that there are two high-pressure bands in patients with HH: one is at the level of the LES and the other is at the level of the crural diaphragm, with no overlap between these two bands 8, 97, 98. Therefore, the synergistic effect of the anti-reflux barrier between the LES and the crural diaphragm is decreased when HH is formed, which significantly reduces the pressure of the LES and weakens the tension of the crural diaphragm 99. In addition, the disappearance of the angle of His and the shortening of the abdominal esophagus also destroys the anti-reflux barrier and ultimately increases the probability of reflux 100** (Figure 3C)**. In addition, HH can also induce the occurrence of TLESR and its frequency is positively correlated with the size of the HH. This may be because HH lowers the threshold of TLESR occurrence 101. However, some studies have suggested that HH does not increase the frequency of TLESR 102, 103. The relationship between the two remains unclear, and further research is needed to clarify this. The mechanism through which HH leads to GERD may also be related to the presence of gastric contents in the hernia sac and reflux of gastric contents when the LES relaxes 8. In addition, another study found that esophageal peristalsis dysfunction, decreased esophageal clearance, increased frequency of acid reflux, esophageal acid exposure time, and reflux symptoms in HH patients were related to the size of the HH. The larger the HH, the longer the acid exposure time, and the more severe the esophagitis 104** (Figure 3C)**. However, there are still many controversies regarding this conclusion. The focus of the debate on whether esophageal peristalsis dysfunction is caused by the HH itself or secondary reflux esophagitis is still unclear, and needs to be explored further by scholars.

Currently, surgery is the main treatment option for HH. Its most important purpose is to close the hiatus defect, while restoring the position and function of the LES, preventing the contents of the abdominal cavity from moving into the thoracic cavity, and minimizing the incidence of reflux 105.

### Esophageal clearance impaired

#### Ineffective esophageal motility (IEM)

Esophageal clearance capacity includes the neutralization of reflux by saliva, weight of food itself, and protrusion of esophageal peristalsis 106. The movement of the esophagus is divided into peristaltic and non-peristaltic contractions. Peristaltic contractions can effectively remove acidic contents of the stomach and duodenum that flow back into the esophagus. However, non-peristaltic contractions are ineffective because of their reduced ability to remove refluxed contents, resulting in prolonged contact with acid and further aggravation of esophageal mucosal damage. Therefore, coordinated and effective peristalsis of the esophageal body is the main mechanism to complete bolus transport and timely removal of reflux. When patients with GERD have esophageal body motor dysfunction, the transmission of bolus in the esophagus is delayed, resulting in pathological characteristics, such as pathological transmission patterns 107. Studies have confirmed that the incidence of IEM in the esophageal body of patients with GERD is 63.95%, and IEM leads to delayed esophageal acid clearance in these patients 108, 109.

In addition, studies have divided patients with GERD into two subgroups: acid reflux and non-acid reflux. When acid reflux events increase, there are significant changes in esophageal motility, which manifest as a decrease in LES pressure, LES length, and distal contractile integral (DCI). It has been suggested that acid exposure may be related to esophageal motility disorders 110. Another study found that, in addition to LES, patients with severe esophagitis also have motor dysfunction in the esophageal body, which causes increased esophageal acid exposure, resulting in esophagitis 111. The mechanism may be that IEM delays the clearance of esophageal acid, which increases the contact time between the acid and the esophageal mucosa. This causes abnormal changes in the esophageal muscle fibers, nerves, and microenvironment, thus leading to chronic inflammation of the esophageal mucosa, and this aggravates acid reflux 112, 113 **(Figure 3C)**. In addition, some scholars used HRM to perform esophageal motility examination in patients with GERD and IEM, before and after Nissen fundoplication, and found that the postoperative esophageal motility function and clinical symptoms of patients significantly improved compared after the procedure compared to the condition before the procedure 114-116. Therefore, scholars speculate that abnormal esophageal motility, especially abnormal esophageal body peristalsis and acid reflux, may be mutually causal. However, another study found that the symptoms of patients with GERD were relieved after the administration of proton pump inhibitors; however, there was no significant improvement in esophageal motility index 108, 117. Therefore, some scholars believe that reflux causes esophageal inflammation, which in turn causes esophageal peristalsis. However, the current mainstream view still tends to be that GERD is caused by upper gastrointestinal tract dysfunction 52. Additionally, with the popularization of HRM technology, more attention has been paid to the GERD mechanism of esophageal motility disorders. IEM, as a newly proposed type of esophageal dynamic disorder, plays an important role in the esophageal dynamic characteristics of GERD, the selection of treatment methods, and the elaboration of its pathogenesis 118-120.

Anti-reflux surgery mainly exerts anti-reflux effect by increasing the barrier function of EGJ, which requires individualized treatment based on the comprehensive evaluation of the patients. Among them, anti-reflux surgery mainly includes Nissen, Toupet and Dor fundoplication 121. Although Nissen fundoplication is the basic operation for anti-reflux surgery, but it is not suitable for patients with severe esophageal motility disorder 122. Studies have shown that IEM is not a risk factor for dysphagia after Nissen fundoplication 114. If IEM patients undergo surgery, the incidence of dysphagia will not increase, but esophageal motor function can improve to a certain extent 114. Recently, it has also been found that patients with IEM who underwent anti-reflux surgery have significantly improved quality of life 123. Therefore, the presence of preoperative IEM should not be a contraindication for Nissen fundoplication, and the key to the optimal outcome of laparoscopic anti-reflux surgery is to carefully evaluate the patient's condition based on the objective data, so as to adjust the surgical approach to provide effective reflux control and improve esophageal clearance 123.

#### Multiple rapid swallowing (MRS)

During esophageal manometry, MRS can be used to assess the presence of a contraction reserve in the esophageal body 124. Among them, MRS can inhibit the central and peripheral nervous system as well as the contraction of esophageal smooth muscle, leading to complete and persistent relaxation of the LES. In addition, the last stage of swallowing during MRS is accompanied by a series of powerful esophageal contractions and contractions after LES relaxation. The normal MRS response not only requires a complete inhibition and excitation mechanism to regulate the coordination between the esophageal body and the LES, but also requires the integrity of the esophageal smooth muscle to respond to the strong stimulation at the end of MRS. Therefore, when the esophageal contractile reserve is present, the MRS peristalsis enhancement ratio is >1 11, 125. One study confirmed that 65% of patients with IEM have an abnormal MRS response 126. Martinucci et al. studied the correlation between the distal contraction integral (DCI) and impedance-pH detection parameters after MRS in 103 patients with heartburn using negative endoscopy. It was also shown that the increase in DCI after MRS was positively correlated with the post-reflux swallow-induced peristaltic wave (PSPW) (*r*=0.626, *p* < 0.01) and negatively correlated with acid exposure time (AET) (*r*=-0.699, *p* < 0.01) 127. Based on this, the Lyon Consensus in 2018 has created a new definition for the diagnosis of GERD 15. Meanwhile, EGJ classification, EGJ-CI, and MRS peristalsis enhancement ratio were taken as new indicators of esophageal motor function, which will help to further promote the advancement in clinical diagnosis and treatment of GERD 15, 128. Since the MRS test has been proved to be an effective indicator in the GERD study 7. Therefore, in the future, the diagnosis of MRS combined with IEM can predict the risk of postoperative dysphagia and guide the selection of treatment modalities.

## Conclusion and current problem

GERD is a complex disease caused by multiple factors, and its pathogenesis has not been fully elucidated. Among them, LES structural abnormalities, hiatal hernia, and esophageal peristalsis dysfunction may play an important role in the pathogenesis of GERD. Although much progress has been made in the pathogenesis of GERD, there are still many problems to be studied, mainly focusing on the following aspects. First, the mechanism of HH involvement in GERD occurrence is still not fully understood. Second, whether esophageal peristalsis dysfunction is primary or secondary to gastroesophageal reflux and whether it is related to the severity of esophagitis, is unknown. Third, there is a lack of effective parameters to comprehensively evaluate the esophageal clearance rate when reflux occurs. Consequently, in-depth research on the etiology and pathogenesis of GERD in future will help to improve the clinicians' understanding of GERD, promote the advancement of diagnosis and treatment methods, and ultimately find a better therapy to improve the quality of life of patients.

## Figures and Tables

**Table 1:** Incidence of GERD symptoms in different geographical locations

	Number of patients (n)	Incidence (%)	95%CI (%)	*P* value
North American	43794	15.4	10.7-20.9	<0.001
South American	24164	17.6	11.0-25.3	<0.001
Middle Eastern	86428	15.0	11.5-19.0	<0.001
South Asian	8864	22.1	11.5-35.0	<0.001
Southeast Asian	58239	7.4	5.0-10.1	<0.001
Australasian	20461	14.1	12.2-16.2	<0.001
Northern European	198686	15.5	13.6-17.5	<0.001
Southern European	19848	21.3	15.8-27.3	<0.001

GERD, gastroesophageal reflux disease.

## Abbreviations

GERD: gastroesophageal reflux disease
LES: lower esophageal sphincter
TLESR: lower esophageal sphincter relaxation
NSAIDs: non-steroidal anti-inflammatory drugs
EGJ: esophagogastric junction
NTS: nucleus of the solitary tract
DMV: dorsal motor nucleus of the vagus nerve
EGJ-CI: esophageal-gastric junction contraction index
HH: hiatal hernia
IEM: ineffective esophageal motility
DCI: distal contractile integral
HRM: high-resolution esophageal manometry
MRS: multiple rapid swallowing
PSPW: post-reflux swallow-induced peristaltic wave
AET: acid exposure time
